# Fibroblast activation protein inhibitor (FAPI) PET for diagnostics and advanced targeted radiotherapy in head and neck cancers

**DOI:** 10.1007/s00259-020-04859-y

**Published:** 2020-05-23

**Authors:** M. Syed, P. Flechsig, J. Liermann, P. Windisch, F. Staudinger, S. Akbaba, S. A. Koerber, C. Freudlsperger, P. K. Plinkert, J. Debus, F. Giesel, U. Haberkorn, S. Adeberg

**Affiliations:** 1 Heidelberg Institute of Radiation Oncology (HIRO), Im Neuenheimer Feld 400, 69120 Heidelberg, Germany; 2 Heidelberg Ion-Beam Therapy Center (HIT), Im Neuenheimer Feld 450, 69120 Heidelberg, Germany; 3 Department of Radiation Oncology, Heidelberg University Hospital, Im Neuenheimer Feld 400, 69120 Heidelberg, Germany; 4 Department of Nuclear Medicine, Heidelberg University Hospital, Im Neuenheimer Feld 400, 69120 Heidelberg, Germany; 5 Department for Radiation Oncology, University Hospital Zurich, Zurich, Switzerland; 6 Department of Oral and Maxillofacial Surgery, Heidelberg University Hospital, Im Neuenheimer Feld 400, 69120 Heidelberg, Germany; 7 Department of Otorhinolaryngology, Head and Neck Surgery, Heidelberg University Hospital, Im Neuenheimer Feld 400, 69120 Heidelberg, Germany; 8 Clinical Cooperation Unit Radiation Oncology, German Cancer Research Center (DKFZ), Im Neuenheimer Feld 280, 69120 Heidelberg, Germany; 9 Clinical Cooperation Unit Nuclear Medicine, DKFZ, Heidelberg, Germany; 10 Translational Lung Research Center Heidelberg (TLRC), German Center for Lung Research (DZL), Heidelberg, Germany

**Keywords:** Fibroblast activation protein, PET-CT, Radiation therapy planning, Head and neck cancer

## Abstract

**Purpose:**

Cancer-associated fibroblasts (CAFs) expressing fibroblast activation protein (FAP) have been associated with the aggressive nature of head and neck cancers (HNCs). These tumours grow diffusely, leading to extremely challenging differentiation between tumour and healthy tissue. This analysis aims to introduce a novel approach of tumour detection, contouring and targeted radiotherapy of HNCs using visualisation of CAFs: PET-CT with ^68^Ga-radiolabeled inhibitors of FAP (FAPI).

**Methods:**

FAPI PET-CT was performed without complications prior to radiotherapy in addition to contrast enhanced CT (CE-CT) and MRI on 14 patients with HNC. First, for tissue biodistribution analysis, volumes of interest were defined to quantify SUV_mean_ and SUV_max_ in tumour and healthy parenchyma. Secondly, using four thresholds of three-, five-, seven- and tenfold increase of FAPI enhancement in the tumour as compared with normal tissue, four different gross tumour volumes (FAPI-GTV) were created automatically. These were compared with GTVs created conventionally with CE-CT and MRI (CT-GTV).

**Results:**

The biodistribution analysis revealed high FAPI avidity within tumorous lesions (e.g. primary tumours, SUV_max_ 14.62 ± 4.44; SUV_mean_ 7.41 ± 2.39). In contrast, low background uptake was measured in healthy tissues of the head and neck region (e.g. salivary glands: SUV_max_ 1.76 ± 0.31; SUV_mean_ 1.23 ± 0.28). Considering radiation planning, CT-GTV was of 27.3 ml, whereas contouring with FAPI resulted in significantly different GTVs of 67.7 ml (FAPI × 3, *p* = 0.0134), 22.1 ml (FAPI × 5, *p* = 0.0419), 7.6 ml (FAPI × 7, *p* = 0.0001) and 2.3 ml (FAPI × 10, p = 0.0001). Taking these significant disparities between the GTVs into consideration, we merged FAPI-GTVs with CT-GTVs. This resulted in median volumes, that were, as compared to CT-GTVs, significantly larger with FAPI × 3 (54.7 ml, + 200.5% relative increase, *p* = 0.0005) and FAPI × 5 (15.0 ml, + 54.9%, *p* = 0.0122). Furthermore, FAPI-GTVs were not covered by CE-CT-based planning target volumes (CT-PTVs) in several cases.

**Conclusion:**

We present first evidence of diagnostic and therapeutic potential of FAPI ligands in head and neck cancer. Larger studies with histopathological correlation are required to validate our findings.

**Electronic supplementary material:**

The online version of this article (10.1007/s00259-020-04859-y) contains supplementary material, which is available to authorized users.

## Introduction

Head and neck cancers (HNC) are the sixth most common malignancy in the world with over 650,000 cases and 330,000 deaths annually [1]. The incidence rates are on the rise over the last years and the patient population is getting younger, especially in the USA and Europe [2].

Radiation therapy (RT) is well established as one of the most important modalities of treating HNC and has immensely contributed to improvements in overall survival of HNC patients. The opportunities of precise RT are growing, e.g. intensity-modulated radiation therapy (IMRT) allows steep gradients. Inescapably, there is a growing necessity for higher precision in diagnostics and differentiation between tumour and adjacent healthy tissue [3]. This is directly relevant for target volume definition for RT and thus decides about tumour recurrence patterns and toxicity to healthy tissue [4]. At the same time, tumour recurrence is often observed within the RT target volume or at its margins [5]. Hence, resistance to RT remains a great challenge.

Most HNCs tend to grow in an invasive and diffuse manner with infiltration of the originating or neighbouring small, delicate and anatomically complex structures such as the otorhinolaryngeal cavities, brain, muscles, bones etc. CT and MRI imaging, despite the application of contrast agents, often fail to demarcate HNC. Positron emission tomography-CT (PET-CT) using ^18^F-fluorodeoxy-d-glucose (^18^F-FDG) tracer is already well recognised for staging, as well as treatment response imaging [4]. However, FDG PET-CT bears several limitations for use in HNC as the technique lacks high contrast. In addition, high glucose uptake and consequently FGD-PET positivity is seen in several crucial healthy tissues such as salivary glands, brain, cervical muscles or lymph nodes [4]. Moreover, false-positive uptake in inflamed peritumour tissue or after surgery and radiotherapy is also very common [6]. With all these weaknesses of FDG PET-CT, it remains difficult to precisely circumscribe the tumours.

Epithelial carcinomas may consist of more than 90% stroma, including also fibroblasts. These carcinoma-associated fibroblasts (CAFs) have recently been identified as key players of tumour invasiveness, progression and therapy resistance [7]. Fibroblast activation protein (FAP) is overexpressed by CAFs of several cancer entities, including HNCs and on the other hand, FAP expression in healthy tissue is relatively low [8].

Thus, visualisation of CAFs using the recently discovered quinoline-based PET tracers, which act as FAP inhibitors (FAPI), is ground-breaking. First in human studies, they have already demonstrated high-contrast tumour imaging using ^68^Ga-FAPI PET-CT [8–12]. In this pioneering study, we are investigating the use of FAPI PET-CT to precisely detect and innovatively delineate HNCs for RT planning.

## Materials and methods

### Patient cohort

This analysis was done using an existing database of 14 HNC patients with age > 18 years (Table 1). They were referred to our Department of Radiation Oncology of the Heidelberg University Hospital, Heidelberg, Germany between July 2017 and August 2018 by their primary otorhinolaryngologists, oral and maxillofacial surgeons or oncologists due to the challenging complexity of the tumours. This complexity required advanced and experimental diagnostic imaging and treatment planning for which we referred them to our collaborating Department of Nuclear Medicine for the FAPI PET-CT.

**Table 1:** Patient characteristics

Total patients	14	
Median age	68.5 (48–83)	
Sex		
Male	12	86%
Female	2	14%
Pre-treatment		
Biopsy only	12	86%
Resection	2	14%
Radiotherapy		100%
Radiotherapy only	6	43%
Radio-chemotherapy	7	50%
Radio-immunotherapy	1	7%
Histology		
Squamous cell carcinoma (SCC)	12	86%
Mucoepidermoid carcinoma	1	7%
Undifferentiated	1	7%

Most of the patients received radiotherapy in definitive setting and only with a prior biopsy for histological confirmation (85.7%). Two patients (14.3%) received additive radiotherapy after surgical resection with macroscopic residual tumour. All patients had histologically confirmed HNCs, whereas squamous cell carcinoma (SCC) was the most common histology (85.7%). Radiotherapy was performed alone or concomitant with chemo- or immunotherapy (Table 1).

### FAPI-PET imaging and biodistribution analysis

All patients gave written informed consent for undergoing ^68^Ga-FAPI PET-CT. The radiopharmaceutical was administered intravenously (80 nmol/GBq) followed by image acquisition 30 min after tracer administration. The PET/CT scans were performed with a Biograph mCT Flow PET/CT-Scanner (Siemens Medical Solutions). A low-dose whole body CT scan (130 keV, 30 mAs, CareDose; reconstructed with a soft-tissue kernel to a slice thickness of 5 mm) was used for attenuation correction and image fusion. A 3-D emission scan (matrix 200 × 200) was performed, subsequently using FlowMotion (Siemens). The emission data was corrected for randoms, scatter and decay. Reconstruction was performed with an ordered subset expectation maximisation (OSEM) algorithm with two iterations/21 subsets and Gauss-filtered to a transaxial resolution of 5 mm at full width at half maximum (FWHM).

Circular volumes of interest were used inside tumour lesions and healthy tissues to quantify the radiotracer biodistribution in patients. This resulted in SUV_max_ and SUV_mean_.

### Target volume delineation

Syngo.via software (VB10B, Siemens Healthineers) was used for target volumetric analyses. For PET-based GTV definition (FAPI-GTV), we compared SUVs of the primary tumour to healthy appearing surrounding tissue. First, we quantified SUV of healthy tissue using region-of-interest method for every patient. This resulted in an individual background value, which was used to define different thresholds of FAPI uptake in the primary tumour. As there is no experience so far in target volume delineation using ^68^Ga-FAPI PET-CT, we used four thresholds of three-, five-, seven- and tenfold increase of FAPI enhancement (SUV_max_) in the tumour as compared with normal tissue to automatically create four different-sized FAPI-GTVs.

These experimental FAPI-GTVs were then correlated with anatomical CT/MR imaging, checked for plausibility and if needed, corrected for false-positive/negative FAPI uptake by two nuclear medicine physicians and two radiation oncologists, experienced and board certified respectively in their fields. Radiation field delimitation is characteristically a subjective task, thus consensus of experts in the field is considered the best standard of reference.

All patients also received contrast-enhanced CT (CE-CT) in combination with an MRI for the conventional radiation treatment planning. GTVs here (CT-GTV) were defined by board-certified radiation oncologists using the latest EORTC guidelines [13] on CT/MR images without the help of PET imaging. Furthermore, by adding a 5-mm margin while respecting anatomical borders, clinical target volumes (CT-CTVs) were created. As a last step, planning target volumes (CT-PTVs) were defined by adding another 5 mm margin to the CT-CTVs. GTVs on their own were often used for applying additional radiation dose (boost) to the tumour.

For better evaluation of discrepancies, we merged FAPI-GTVs with CT-GTVs and compared the merged GTVs with CT-GTVs. Lastly, we also compared FAPI-GTVs with CE-CT-PTVs as PTV is the last boundary that provides therapeutic radiation dose for tumour control.

### Statistics

Wilcoxon matched-pairs signed rank test was used to check for significant differences (*p* < 0.05).

## Results

### Radiopharmaceutical safety

All patients tolerated ^68^Ga-FAPI PET-CT without any complication. No symptoms were reported during injection and the 1.5-h follow-up.

### Biodistribution analysis

**Fig. 1:**
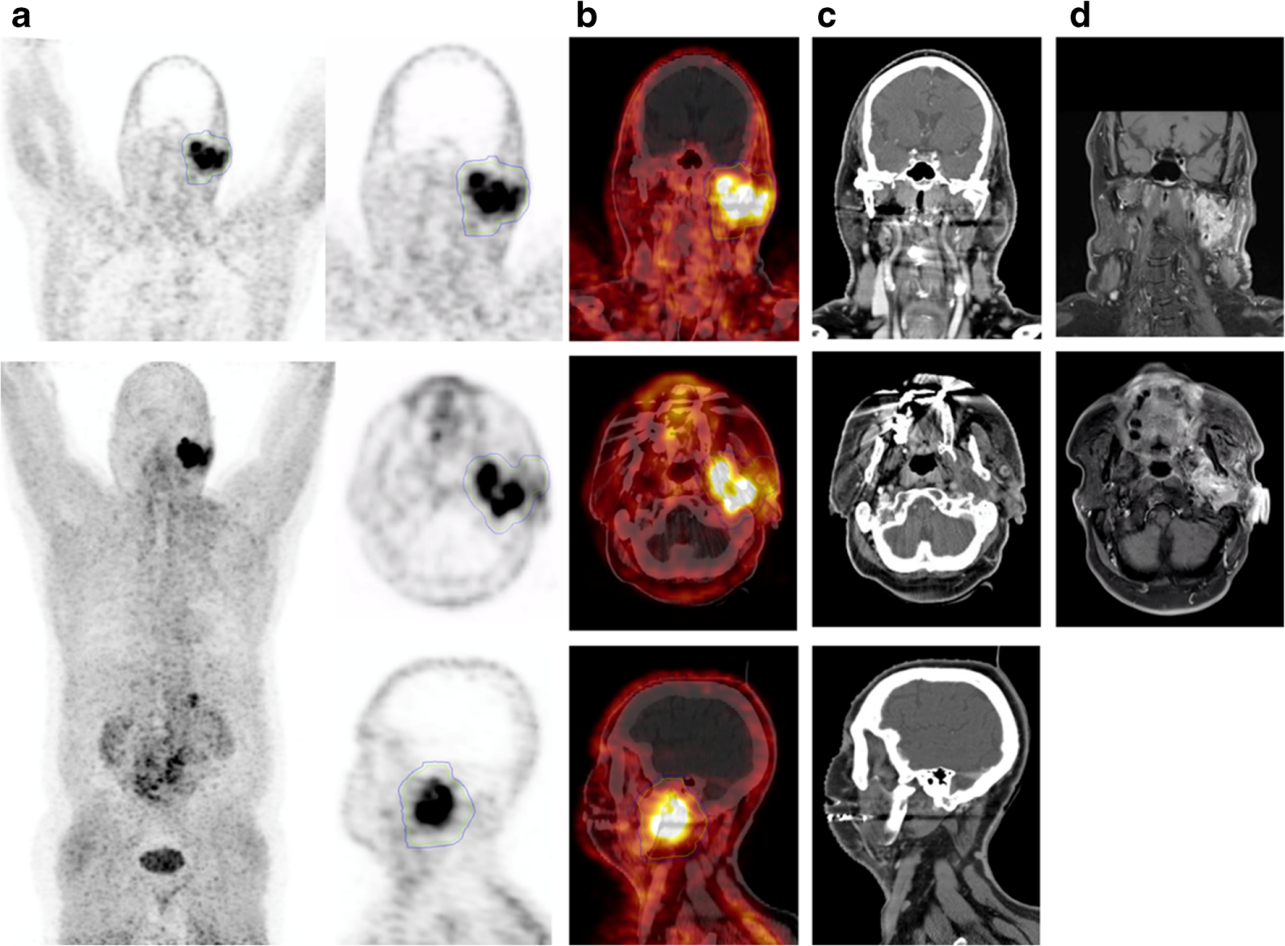
Example of a 64-year-old male patient with mucoepidermoid carcinoma of the left parotid gland receiving FAPI-PET CT and radiation treatment. **a**, **b** MIP and CT-fused FAPI-PET images showing precise tracer uptake by the parotid carcinoma and very low background noise. **c**, **d** Conventional CE-CT and CE-MR imaging showing diffuse tumour infiltration making differentiation between tumour and healthy tissue enormously difficult and subjective. Abbreviations: CE, contrast enhanced; FAPI-PET, ^68^Ga-fibroblast activation protein inhibitor PET-CT

**Fig. 2:**
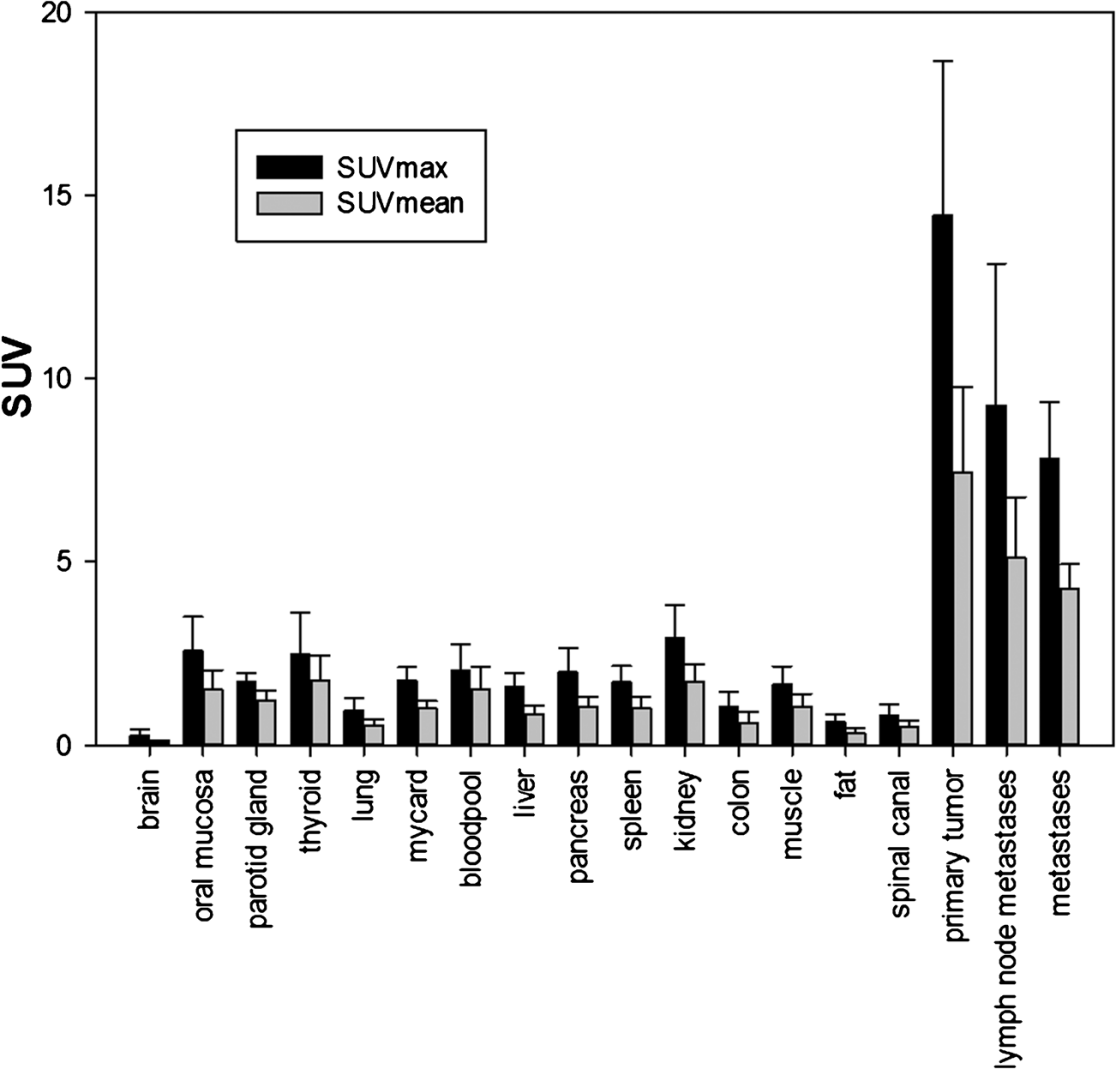
Biodistribution analysis of ^68^Ga-FAPI PET-CT in whole body imaging with maximum and mean standard uptake values (SUV_max/mean_) of the tumour, metastases and healthy tissues. Prominently high SUVs in tumorous lesions as compared with healthy tissues are seen

In a first step, we performed biodistribution analyses for ^68^Ga-FAPI for evaluation of imaging resolution quality and standardisation. SUV_max_ and SUV_mean_ were used for this purpose (Figs. 1 and 2).

The highest activity concentration was measured in the primary tumour (SUV_max_ 14.62 ± 4.44; SUV_mean_ 7.41 ± 2.39), followed by lymph node metastases (SUV_max_ 9.42 ± 5.72; SUV_mean_ 5.08 ± 2.12) and bone metastases (SUV_max_ 7.51 ± 1.75; SUV_mean_ 4.1 ± 0.9). Compared with the primary tumour and the locoregional lymph node metastases, considerably low background uptake was measured in the head and neck region, namely the brain (SUV_max_ 0.30 ± 0.22; SUV_mean_ 0.06 ± 0.03), oral mucosa (SUV_max_ 2.57 ± 1.00; SUV_mean_ 1.55 ± 0.55), muscles (SUV_max_ 1.76 ± 0.6; SUV_mean_ 1.09 ± 0.39) and salivary glands (SUV_max_ 1.76 ± 0.31; SUV_mean_ 1.23 ± 0.28).

### Automated target volume delineation

**Fig. 3:**
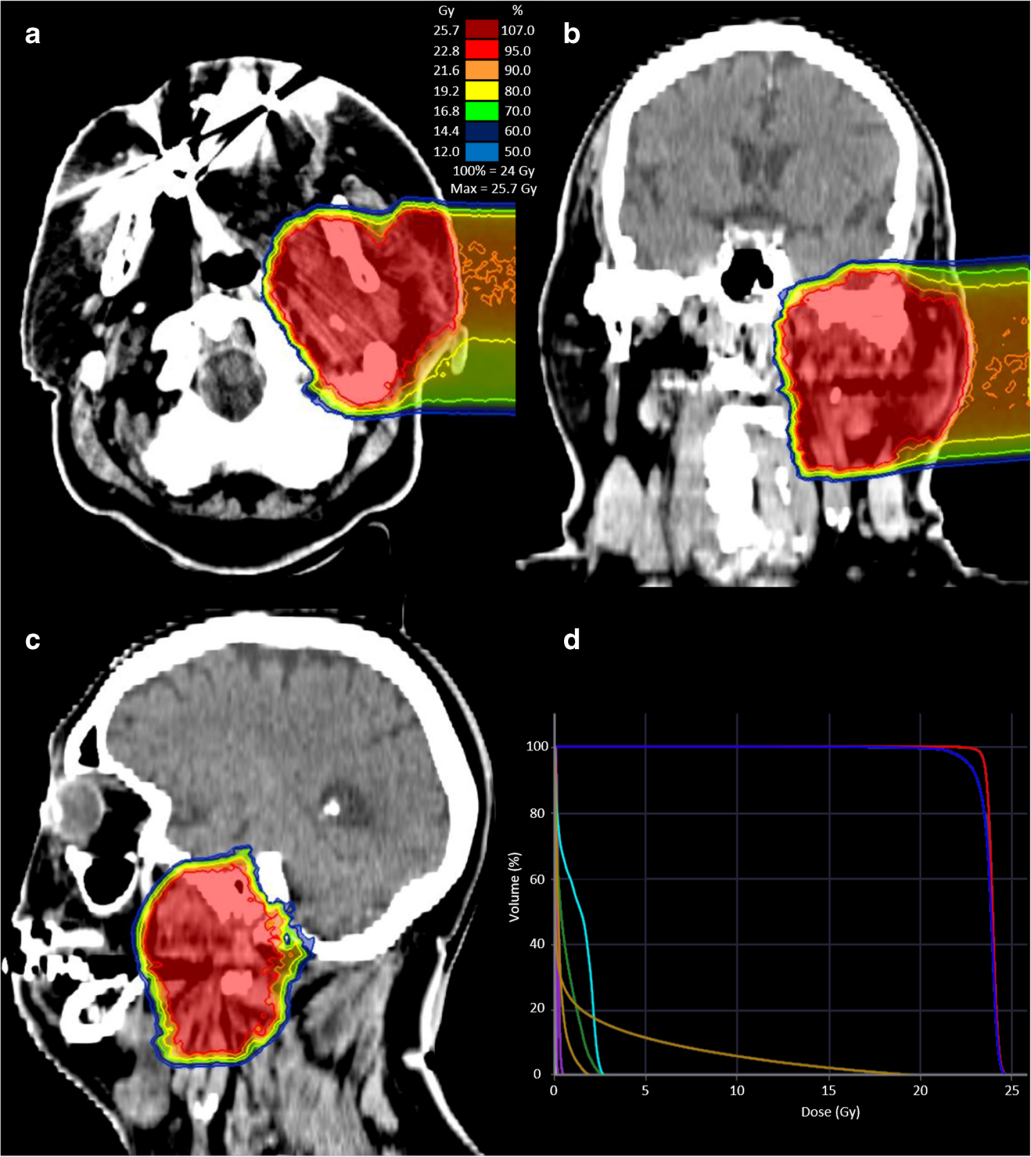
Radiation treatment plan of the patient presented in Fig. 1 with: **a** axial, **b** coronal and **c** sagittal dose distribution and the **d** dose-volume histogram. After partial resection of the tumour, the patient received IMRT with photons with a total dose of 50 Gy in 25 fractions, followed by a carbon-ion boost on the GTV with a total dose of 24 Gy (RBE) in 8 fractions. Abbreviations: MIP, maximum intensity projection; Gy, Gray; IMRT, intensity-modulated radiotherapy; RBE, relative biological effectiveness

**Fig. 4:**
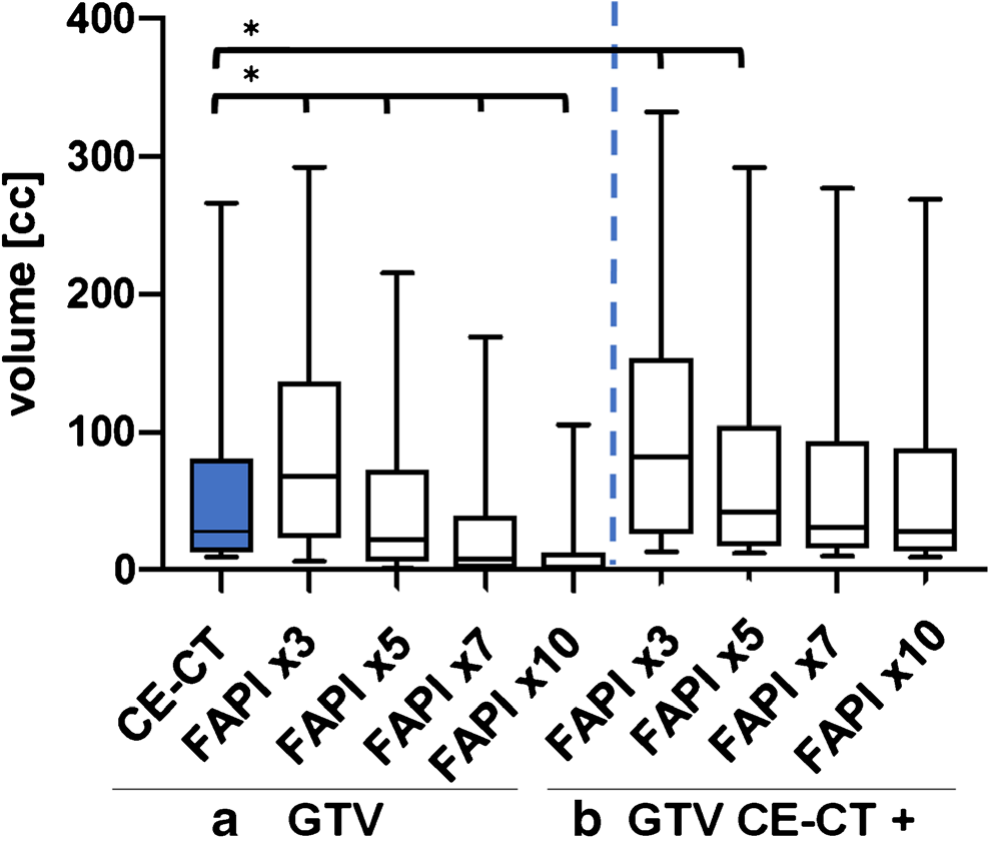
**a** Comparison of GTVs based on CE-CT/MRI and FAPI with four different thresholds in relation to uptake in the healthy tissue. Throughout, significantly different FAPI-based GTVs are seen. **b** CT-GTVs fused with FAPI-GTVs showing significant increase in volumes with FAPI × 3 and × 5 as compared with CE-CT. *Significant. Abbreviations: GTV, gross tumour volume; CT-GTV, GTV based on CT/MRI; FAPI-GTV, GTV based on ^68^Ga-FAPI PET-CT

These findings were directly translated into radiation treatment planning for tumour volume delineation (an examplary RT plan is shown in Fig. 3). Conventional CT-GTVs showed a median volume of 27.3 ml (range 9.1–266.5 ml). On the other hand, contouring with ^68^Ga-FAPI PET-CT resulted in significantly different GTVs in all SUV thresholds of FAPI × 3: 67.7 ml (*p* = 0.013, range 6.0–292.7); FAPI × 5: 22.1 ml (*p* = 0.042, range 0.9–215.5); FAPI × 7: 7.6 ml (*p* = 0.0001, range 0.0–168.9); and FAPI ×10: 2.3 ml (*p* = 0.0001, 0.0–105.3) (see also Fig. 4).

### Comparison of FAPI-GTVS and CT-GTVs and CT-PTVs

Taking these significant disparities between CT-GTVs and FAPI-GTVs into consideration, we merged FAPI-GTVs with CT-GTVs. This resulted in median volumes that were, as compared with CT-GTVs, significantly larger with FAPI × 3 (+ 54.7 ml, + 200.5% relative increase, *p* = 0.0005) and FAPI × 5 (+ 15.0 ml, + 54.9%, *p* = 0.012) (Fig. 4).

In a next step, to see whether FAPI-GTVs were included in the radiation treatment plan or not, we added them to CE-CT-based planning target volumes (CT-PTVs). Several patients showed FAPI-avid primary tumour regions that were not covered by CT-PTV but were a part of the FAPI-GTV.

## Discussion

We achieved high-contrast images with ^68^Ga-FAPI PET-CT due to very specific and high tracer uptake in tumours and low background noise. Especially in the refine head and neck area we saw very low uptake in healthy parenchyma adjacent to the tumour, including in the brain, oral/laryngeal mucosa, salivary glands (e.g. parotid gland) and muscles (Figs. 1 and 3). In addition, in the context of peritumoural inflammation or status post-resection or biopsy, no false-positive uptake was seen adjacent to the tumours. Hence, we could emphasise current discoveries about the high sensitivity and specificity of FAPI-PET [8, 9].

Considering these findings and in light of the biological background of FAPI-PET, based on visualisation of CAFs, new dimensions for targeted therapy were revealed. We implemented this innovative technology in target volume delineation for radiation therapy and could automatically generate biological target volumes based on different experimental tumour-to-healthy tissue FAPI-SUVs ratios (Fig. 3). The alternate method that uses %-SUV_max_ threshold produced similar GTVs: e.g. 3-fold background cut-off is equivalent to 20–25% SUV_max_ and 5-fold background cut-off is equivalent to 40–50% SUV_max_. For validation, board certified specialists for nuclear medicine and radiation oncology worked together in 14 oncologically challenging cases of HNC. With the automated, FAPI-based contouring methodology, we aimed to find a universal SUV_max_ threshold for tumours (based on individual SUV_max_ of healthy tissue) that radiation oncologists can easily use to contour HNCs automatically and if needed, manually adjust in comparison with anatomical imaging.

Surprisingly, all FAPI-based GTVs were significantly different than the conventional CT-GTVs (Fig. 4). In consensus with our team of experienced nuclear medicine physicians and radiation oncologists, FAPI × 3 threshold emerged to be ideal for precise tumour detection and for sparing healthy tissue. The three other thresholds instead appeared to deliver insufficiently small GTVs where parts of tumour were omitted. However, merging FAPI-GTV with CT-GTV revealed that even FAPI × 5, FAPI × 7 and FAPI × 10 thresholds contained vital, FAPI-avid tumour extents that were not part of CT-GTVs. The merged FAPI × 3 and FAPI × 5 GTVs for instance were significantly larger by 200.5 and 54.9%, respectively. This finding was further highlighted when we saw parts of FAPI-GTV not even encompassed in CT-PTV in several patients. Hence, with conventional radiation treatment planning, these vital and possibly more aggressive parts of the tumours (see below) would have received insufficient radiation dose, as they were not included in the GTV boost or would have received no radiation at all as they were not included in the PTV.

Our findings have substantial implications as tumour recurrence is seen in 15 to 50% of patients with HNC [5, 14]. The main causes of recurrence have been reported as radiation resistance in tumour cells or inadequate initial treatment such as insufficient radiation dose, volume or fractionation [15]. Furthermore, heterogeneity in intra-tumour malignancy has been disregarded in radiation dose application resulting in possible radiation under- and overdosing. This leads to the many cases of tumour recurrence within GTV [16]. Inter-physician variability in radiation target volume definition is another major source of uncertainty in HNC treatment. Deficiency in reproducibility and inconsistency of manual target volume delineation has direct consequences for tumour recurrence [17]. All of these causes behind tumour relapse are directly associated with increased mortality and poor survival rates [5, 14]. Thus, recurrent disease remains the main obstacle to long-term survival. In addition, salvage treatment options are often limited because of multiple reasons, including restrictions due to first therapy; higher morbidity caused by the retreatment, especially re-irradiation and commonly the multifocal nature of recurrent disease [18].

On the other hand, long-term survival rates of patients with HNC have improved over the years. The predominant reasons are early detection of tumours, improved treatment options and a shift in tumour aetiology. From 1988 to 2004, an increase of up to 225% has been reported for human papilloma virus (HPV) associated tumours. These tumours develop in younger patients and show significantly improved survival rates than their counterparts which emerge in older patients and are associated with smoking and drinking [19, 20]. Hence, it is even more essential to reduce late toxicities by sparing healthy tissue during RT.

All of the above data suggest the inevitable necessity to improve the initial radiation therapy plans. In comparison with conventional anatomical CT and MR imaging, ^18^F-FDG PET-CT has shown the possibility to detect FDG-avid primary tumours, lymph node metastases and distant metastases with high sensitivity. Hence, target volume sizes can possibly be decreased by only including involved regions [21]. This upgrade has several limitations as FDG PET-CT is less specific due to false positive findings. Thus, especially in the head and neck region, it is unable to precisely assess local tumour spread in correlation with delicate and complex peritumoural structures [4].

This preliminary study with 14 patients cannot sufficiently calculate sensitivity, specificity and accuracy of the new tracer. Yet, FAPI PET-CT crystallises as a promising candidate for effective and non-invasive visualisation of specifically the tumour stroma, which can make up to 90% of the tumour and mainly consists of CAFs [7]. CAFs with especially the subtypes expressing FAP have been reported not only to physically support cancer cells but also to be key players of tumour angiogenesis. They produce several growth factors such as vascular endothelial growth factor (VEGF) or fibroblast growth factor (FGF). These factors lead to tumour formation, proliferation and metastasis [22]. In addition, resistance of many cancer cells to radiation and chemotherapy is also contributed by CAFs [23, 24]. Recent studies have further revealed that CAFs with high FAP expression not only lead to resistance to the body’s own immune response but also to resistance to immune-checkpoint inhibitor therapy [25].

Hence, directly targeting CAFs which are made visible by FAPI-PET, with a radiation boost emerges as a new perspective of treatment not only because the cancer is more accurately targeted but also because the precise elimination of CAFs can sensitise the entire tumour to radiation, chemo-immunotherapy and the body’s own immune system [7, 25]. Moreover, excluding FAPI-negative areas from target volumes would spare toxicity.

The next promising advancement appears in the knowledge that intra-tumoural uptake-intensity of PET tracers reflects the grade of malignancy [26, 27]. Molecular biological analyses have shown that higher density of stroma with CAFs and FAP overexpression within the tumour is associated with increased tumour migration, invasion and therapy resistance [7, 28, 29]. Furthermore, higher FAP expression is seen in invasive areas of tumours such as tumour borders and microscopic tumour cell protrusions, also known as invadopodia [30, 31]. These elements and areas of higher malignancy are linked with increased therapy resistance, likelihood of tumour recurrence and consequently worse survival. FAPI-PET non-invasively and conveniently visualises this valuable biologic information through different SUVs and can subsequently enable innovative, precise and tailored radiation dose escalation or de-escalation plans for tumour subvolumes, also known as dose painting.

Another potential of FAPI imaging lies in the update of early response evaluation during and after therapy. Radiotherapy induces biological and molecular changes in the tumour microenvironment which can be visualised by PET tracers [32, 33]. Thanks to this information, plans could be adapted during treatment and follow-up regimens could be personalised.

The limitation for dose painting and therapy adaptation is the finite resolution of PET which might not mirror the microregional spatial distribution of cells in the tumour [34]. Hence, further studies with histopathological gold standard are warranted after this hypothesis, generating analysis. It is essential to evaluate the impact of FAPI PET-CT in the staging of head and neck tumours and to observe the rate of false-positive and false-negative imaging findings with this novel radioligand as compared with the above-mentioned imaging modalities and histology. Especially, intra-individual comparison between FAPI PET-CT and the current standard in oncology, FDG-PET would show if FAPI-PET is truly non-inferior or even superior.

Due to limited experience with therapeutic implication of FAPI-PET for radiotherapy of HNCs, target volume delineation should be performed in combination of anatomical imaging and in close cooperation of experienced nuclear medicine physicians and radiation oncologists. Further studies with higher patient numbers are needed to evaluate the optimal threshold not only to specify precise tumour volume but also the healthy tissue volume to reduce side effects of radiation therapy. Optimally, this advancement would enable us to automatically and uniformly delineate tumour volumes with lower inter-physician variability.

Further studies should also include pattern of failure analyses and verify the survival impact of individual dose adaptation of tumour subvolumes based on FAPI-PET, particularly when using advanced radiation techniques.

## Conclusion

We present first evidence of diagnostic and therapeutic potential of FAPI-PET CT in head and neck cancer. Larger studies with histopathological correlation are required to validate our findings.

## Electronic supplementary material

Fig S1 Example of a 52-year-old male patient with HPV-positive nasopharyngeal squamous cell carcinoma with cervical lymph node metastases: **a** MIP images of the whole body FAPI-PET scan; **b** CT-fused FAPI-PET images, **c** conventional CT images and **d** conventional MR images showing the primary tumour and the cervical lymph node metastasis. After biopsy with confirmation of the diagnosis, the patient received five neoadjuvant cycles of chemotherapy with carboplatin/paclitaxel weekly followed by definitive radiotherapy. The radiation treatment consisted of an initial IMRT photon plan with 56 Gy in 28 fractions to the nasopharynx and the cervical lymph node regions. It was followed by an additional carbon-ion boost to the GTVs of the primary tumour and the lymph node metastases with 18 Gy (RBE) in 6 fractions. Abbreviations: HPV, human papilloma virus; MIP, maximum intensity projection; Gy, Grey; IMRT, intensity-modulated radiotherapy; RBE, relative biological effectiveness (PNG 2.48 mb)

## List of abbreviations

^18^F-FDG: ^18^F-fluorodeoxy-d-glucose
^68^Ga-FAPI: ^68^Ga-fibroblast activation protein inhibitor
CE-CT: Contrast-enhanced computer tomography
CT: Computer tomography
EORTC: European Organisation for Research and Treatment of Cancer
FAP: Fibroblast activation protein
GTV: Gross tumour volume
HNC: Head and neck cancer
HPV: Human papilloma virus
IMRT: Intensity modulated radiotherapy
MRI: Magnetic resonance imaging
PET: Positron emission tomography
PTV: Planning target volume
SCC: Squamous cell carcinoma
SUV: Standardised uptake value

## References

[1] Bray F, Ferlay J, Soerjomataram I, Siegel RL, Torre LA, Jemal A (2018). Global cancer statistics 2018: GLOBOCAN estimates of incidence and mortality worldwide for 36 cancers in 185 countries. CA Cancer J Clin.

[2] Boscolo-Rizzo P, Zorzi M, Del Mistro A, Da Mosto MC, Tirelli G, Buzzoni C (2018). The evolution of the epidemiological landscape of head and neck cancer in Italy: is there evidence for an increase in the incidence of potentially HPV-related carcinomas?. PLoS One.

[3] Alterio D, Marvaso G, Ferrari A, Volpe S, Orecchia R, Jereczek-Fossa BA. Modern radiotherapy for head and neck cancer. Semin Oncol. 2019. 10.1053/j.seminoncol.2019.07.002. 10.1053/j.seminoncol.2019.07.002 31378376

[4] Castaldi P, Leccisotti L, Bussu F, Micciche F, Rufini V (2013). Role of (18)F-FDG PET-CT in head and neck squamous cell carcinoma. Acta Otorhinolaryngol Ital.

[5] Leeman JE, Li JG, Pei X, Venigalla P, Zumsteg ZS, Katsoulakis E (2017). Patterns of treatment failure and postrecurrence outcomes among patients with locally advanced head and neck squamous cell carcinoma after chemoradiotherapy using modern radiation techniques. JAMA Oncol.

[6] Hentschel M, Appold S, Schreiber A, Abramyuk A, Abolmaali N, Kotzerke J (2009). Serial FDG-PET on patients with head and neck cancer: implications for radiation therapy. Int J Radiat Biol.

[7] Wang Z, Tang Y, Tan Y, Wei Q, Yu W (2019). Cancer-associated fibroblasts in radiotherapy: challenges and new opportunities. Cell Commun Signal.

[8] Kratochwil C, Flechsig P, Lindner T, Abderrahim L, Altmann A, Mier W (2019). (68)Ga-FAPI PET/CT: tracer uptake in 28 different kinds of cancer. J Nucl Med.

[9] Giesel FL, Kratochwil C, Lindner T, Marschalek MM, Loktev A, Lehnert W (2019). (68)Ga-FAPI PET/CT: biodistribution and preliminary dosimetry estimate of 2 DOTA-containing FAP-targeting agents in patients with various cancers. J Nucl Med.

[10] Lindner T, Loktev A, Altmann A, Giesel F, Kratochwil C, Debus J (2018). Development of quinoline-based theranostic ligands for the targeting of fibroblast activation protein. J Nucl Med.

[11] Loktev A, Lindner T, Mier W, Debus J, Altmann A, Jager D (2018). A tumor-imaging method targeting cancer-associated fibroblasts. J Nucl Med.

[12] Rohrich M, Loktev A, Wefers AK, Altmann A, Paech D, Adeberg S, et al. IDH-wildtype glioblastomas and grade III/IV IDH-mutant gliomas show elevated tracer uptake in fibroblast activation protein-specific PET/CT. Eur J Nucl Med Mol Imaging. 2019. 10.1007/s00259-019-04444-y. 10.1007/s00259-019-04444-y 31388723

[13] Gregoire V, Evans M, Le QT, Bourhis J, Budach V, Chen A (2018). Delineation of the primary tumour clinical target volumes (CTV-P) in laryngeal, hypopharyngeal, oropharyngeal and oral cavity squamous cell carcinoma: AIRO, CACA, DAHANCA, EORTC, GEORCC, GORTEC, HKNPCSG, HNCIG, IAG-KHT, LPRHHT, NCIC CTG, NCRI, NRG oncology, PHNS, SBRT, SOMERA, SRO, SSHNO, TROG consensus guidelines. Radiother Oncol.

[14] Brockstein B, Haraf DJ, Rademaker AW, Kies MS, Stenson KM, Rosen F (2004). Patterns of failure, prognostic factors and survival in locoregionally advanced head and neck cancer treated with concomitant chemoradiotherapy: a 9-year, 337-patient, multi-institutional experience. Ann Oncol.

[15] McDonald MW, Lawson J, Garg MK, Quon H, Ridge JA, Saba N (2011). ACR appropriateness criteria retreatment of recurrent head and neck cancer after prior definitive radiation expert panel on radiation oncology-head and neck cancer. Int J Radiat Oncol Biol Phys.

[16] Soto DE, Kessler ML, Piert M, Eisbruch A (2008). Correlation between pretreatment FDG-PET biological target volume and anatomical location of failure after radiation therapy for head and neck cancers. Radiother Oncol.

[17] Cardenas CE, McCarroll RE, Court LE, Elgohari BA, Elhalawani H, Fuller CD (2018). Deep learning algorithm for auto-delineation of high-risk oropharyngeal clinical target volumes with built-in dice similarity coefficient parameter optimization function. Int J Radiat Oncol Biol Phys.

[18] Ho AS, Kraus DH, Ganly I, Lee NY, Shah JP, Morris LG (2014). Decision making in the management of recurrent head and neck cancer. Head Neck.

[19] Chaturvedi AK, Engels EA, Pfeiffer RM, Hernandez BY, Xiao W, Kim E (2011). Human papillomavirus and rising oropharyngeal cancer incidence in the United States. J Clin Oncol.

[20] Ryerson AB, Peters ES, Coughlin SS, Chen VW, Gillison ML, Reichman ME (2008). Burden of potentially human papillomavirus-associated cancers of the oropharynx and oral cavity in the US, 1998-2003. Cancer..

[21] Wong WL, Sonoda LI, Gharpurhy A, Gollub F, Wellsted D, Goodchild K (2012). 18F-fluorodeoxyglucose positron emission tomography/computed tomography in the assessment of occult primary head and neck cancers—an audit and review of published studies. Clin Oncol (R Coll Radiol).

[22] Erez N, Truitt M, Olson P, Arron ST, Hanahan D (2010). Cancer-associated fibroblasts are activated in incipient neoplasia to orchestrate tumor-promoting inflammation in an NF-kappaB-dependent manner. Cancer Cell.

[23] Yoshida GJ, Azuma A, Miura Y, Orimo A. Activated fibroblast program orchestrates tumor initiation and progression; molecular mechanisms and the associated therapeutic strategies. Int J Mol Sci. 2019;20. 10.3390/ijms20092256. 10.3390/ijms20092256 PMC6539414 31067787

[24] Shiga K, Hara M, Nagasaki T, Sato T, Takahashi H, Takeyama H (2015). Cancer-associated fibroblasts: their characteristics and their roles in tumor growth. Cancers (Basel).

[25] Liu T, Han C, Wang S, Fang P, Ma Z, Xu L (2019). Cancer-associated fibroblasts: an emerging target of anti-cancer immunotherapy. J Hematol Oncol.

[26] Hatt M, Majdoub M, Vallieres M, Tixier F, Le Rest CC, Groheux D (2015). 18F-FDG PET uptake characterization through texture analysis: investigating the complementary nature of heterogeneity and functional tumor volume in a multi-cancer site patient cohort. J Nucl Med.

[27] Hatt M, Tixier F, Pierce L, Kinahan PE, Le Rest CC, Visvikis D. Characterization of PET/CT images using texture analysis: the past, the present... any future? Eur J Nucl Med Mol Imaging 2017;44:151–165. doi:10.1007/s00259-016-3427-0. 10.1007/s00259-016-3427-0 PMC5283691 27271051

[28] Gascard P, Tlsty TD (2016). Carcinoma-associated fibroblasts: orchestrating the composition of malignancy. Genes Dev.

[29] Henriksson ML, Edin S, Dahlin AM, Oldenborg PA, Oberg A, Van Guelpen B (2011). Colorectal cancer cells activate adjacent fibroblasts resulting in FGF1/FGFR3 signaling and increased invasion. Am J Pathol.

[30] Ghersi G, Dong H, Goldstein LA, Yeh Y, Hakkinen L, Larjava HS (2002). Regulation of fibroblast migration on collagenous matrix by a cell surface peptidase complex. J Biol Chem.

[31] Sandberg TP, Stuart M, Oosting J, Tollenaar R, Sier CFM, Mesker WE (2019). Increased expression of cancer-associated fibroblast markers at the invasive front and its association with tumor-stroma ratio in colorectal cancer. BMC Cancer.

[32] Troost EG, Schinagl DA, Bussink J, Oyen WJ, Kaanders JH (2010). Clinical evidence on PET-CT for radiation therapy planning in head and neck tumours. Radiother Oncol.

[33] Cliffe H, Patel C, Prestwich R, Scarsbrook A (2017). Radiotherapy response evaluation using FDG PET-CT-established and emerging applications. Br J Radiol.

[34] Newbold K, Powell C (2012). PET/CT in radiotherapy planning for head and neck cancer. Front Oncol.

