# Supplementary figures and images for: Fibroblast activation protein inhibitor (FAPI) PET for diagnostics and advanced targeted radiotherapy in head and neck cancers
Source: Eur J Nucl Med Mol Imaging. 2020 May 23;47(12):2836–45. doi: 10.1007/s00259-020-04859-y (PMC7567680; doi:10.1007/s00259-020-04859-y)

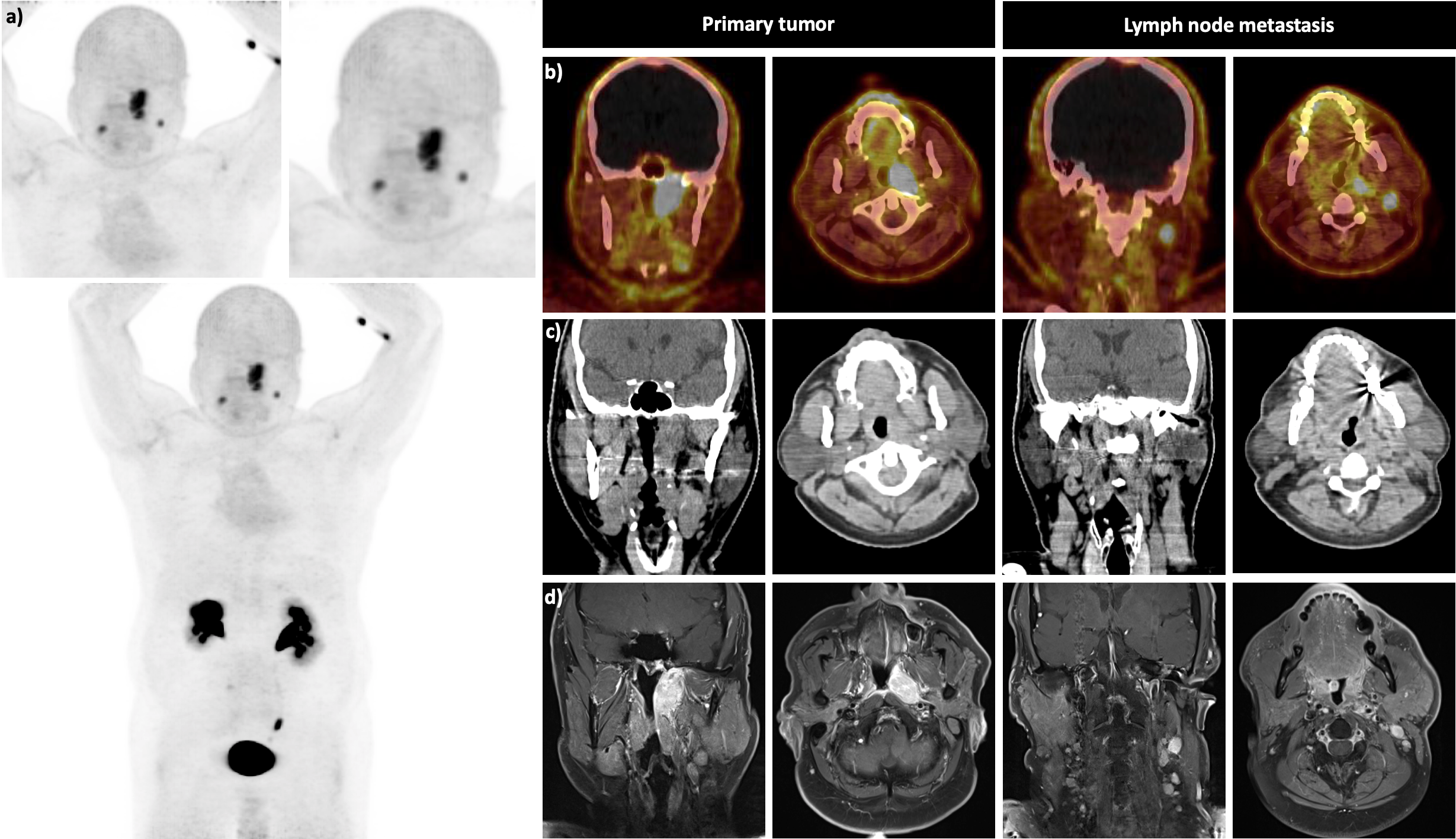

Supplement: Supplementary file 1 — Example of a 52-year-old male patient with HPV-positive nasopharyngeal squamous cell carcinoma with cervical lymph node metastases: a MIP images of the whole body FAPI-PET scan; b CT-fused FAPI-PET images, c conventional CT images and d conventional MR images showing the primary tumour and the cervical lymph node metastasis. After biopsy with confirmation of the diagnosis, the patient received five neoadjuvant cycles of chemotherapy with carboplatin/paclitaxel weekly followed by definitive radiotherapy. The radiation treatment consisted of an initial IMRT photon plan with 56 Gy in 28 fractions to the nasopharynx and the cervical lymph node regions. It was followed by an additional carbon-ion boost to the GTVs of the primary tumour and the lymph node metastases with 18 Gy (RBE) in 6 fractions. Abbreviations: HPV, human papilloma virus; MIP, maximum intensity projection; Gy, Grey; IMRT, intensity-modulated radiotherapy; RBE, relative biological effectiveness (PNG 2.48 mb) [file 259_2020_4859_MOESM1_ESM.png]
